# Ectopic Expression of a Wheat R2R3-Type MYB Gene in Transgenic Tobacco Enhances Osmotic Stress Tolerance via Maintaining ROS Balance and Improving Root System Architecture

**DOI:** 10.3390/biology13020128

**Published:** 2024-02-18

**Authors:** Omar Azab, Walid Ben Romdhane, Salah El-Hendawy, Abdelhalim Ghazy, Adel M. Zakri, Ahmed M. Abd-ElGawad, Abdullah Al-Doss

**Affiliations:** Plant Production Department, College of Food and Agriculture Sciences, King Saud University, P.O. Box 2460, Riyadh 11451, Saudi Arabia; mosalah@ksu.edu.sa (S.E.-H.); aghazy@ksu.edu.sa (A.G.); azakri@ksu.edu.sa (A.M.Z.); aibrahim2@ksu.edu.sa (A.M.A.-E.); aaldoss@ksu.edu.sa (A.A.-D.)

**Keywords:** osmotic stress, root system architecture, R2R3-type MYB, wheat

## Abstract

**Simple Summary:**

Agriculture is the sector where water scarcity has the greatest impact; it alters the pattern of plant growth and productivity. Therefore, understanding drought-stress response mechanisms and the isolation of key genes involved in these mechanisms can help breeders to select drought-tolerant wheat varieties to ensure the sustainability of cereal production. Herein, we isolated and functionally characterized the potential of a wheat R2R3-type MYB gene called *TaMYB20* to improve root system architecture using a genetic engineering approach. The *TaMYB20-*overexpressing tobacco plants exhibited enhanced tolerance to extended osmotic stress associated with maintenance of ROS balance, lower ion leakage, high relative water content, upregulation of auxin-related genes expression, and improving root system architecture. Overall, these findings provide insight into the potential of *TaMYB20* gene to improve drought tolerance, and it could be a potentially useful candidate gene for engineering drought tolerance in cultivated plants.

**Abstract:**

Water scarcity is a critical cause of plant yield loss and decreased quality. Manipulation of root system architecture to minimize the impact of water scarcity stresses may greatly contribute towards an improved distribution of roots in the soil and enhanced water and nutrient uptake abilities. In this study, we explored the potential of *TaMYB20* gene, a wheat gene belonging to the R2R3-MYB transcription factor family, to improve root system architecture in transgenic tobacco plants. The full-length *TaMYB20* gene was isolated from *Triticum aestivum*.cv. Sakha94 and used to produce genetically engineered tobacco plants. The transgenic plants exhibited enhanced tolerance to extended osmotic stress and were able to maintain their root system architecture traits, including total root length (TRL), lateral root number (LRN), root surface area (RSa), and root volume (RV), while the wild-type plants failed to maintain the same traits. The transgenic lines presented greater relative water content in their roots associated with decreased ion leakage. The oxidative stress resulted in the loss of mitochondrial membrane integrity in the wild-type (WT) plants due to the overproduction of reactive oxygen species (ROS) in the root cells, while the transgenic lines were able to scavenge the excess ROS under stressful conditions through the activation of the redox system. Finally, we found that the steady-state levels of three *PIN* gene transcripts were greater in the *TaMYB20*-transgenic lines compared to the wild-type tobacco. Taken together, these findings confirm that *TaMYB20* is a potentially useful gene candidate for engineering drought tolerance in cultivated plants.

## 1. Introduction

Plant growth is predominantly determined by adverse environmental factors such as drought and salinity, which alter the pattern of normal growth and productivity. Plants activate many complex networks of signal transduction to regulate the expression of stress-related genes to allow them to adapt their environments [1,2]. The MYB transcription factors (TFs) family—one of the largest families in plants—is characterized by the conserved MYB DNA-binding domains, which divide the super family into four subgroups according to the number of MYB-domain repeats (R). These proteins typically contain 1–4 R motifs: MYB1R, R2R3-MYB, MYB3R, and 4R-MYB [3]. Among these four motifs, R2R3-MYB protein is considered the most important subgroup because it contains the most members. Several studies have shown that MYB proteins participate in numerous biological processes in plants in response to abiotic stresses, such as AtMYB96 in drought and freezing tolerance and auxin signaling pathways [4,5], AtMYB44 and AtMYB60 in regulating stomatal movement through drought stress [6,7], and AtMYB14 and AtMYB15, which participate in plant adaptation to freezing [8,9]. The overexpression of GbMYB5 in *Gossypium barbadense* (cotton) and *Nicotiana tabacum* (tobacco) plants confers drought tolerance [10]. The R2R3-type MYB gene SlMYB102 confers salt tolerance to transgenic tomatoes comparing to the wild-type plants [11], whereas the novel R2R3-MYB gene IbMYB116 isolated from sweet potato enhances drought tolerance in transgenic Arabidopsis through the JA signaling pathway [12]. AtMYB20 enhances salt-stress tolerance [13]. The OsMYB22 gene is involved in the tolerance of various stresses such as salt, freezing, and dehydration [14], while OsMYB91 coordinates salt-stress tolerance and plant growth [15]. The overexpression of *Glycine max* (soybean) GmMYB84 in transgenic Arabidopsis confers salt tolerance [16]. Few MYB genes have been characterized in wheat, although many of those genes have been identified; for example, the expression of TaMyb1 in wheat roots can be induced by abiotic stresses [17]. The TaMYB19 confers enhanced tolerance to abiotic stress [18]. Moreover, the TaMyb1D was first cloned from wheat and shown to confer drought and oxidative stress tolerance to transgenic tobacco through the regulation of flavonoid biosynthesis [19]. In common wheat, the expression of R2R3-MYB TaMYB20 was upregulated in the roots, stems, and leaves under abiotic stress conditions, suggesting its involvement in salt and drought responses in plants [20]. Given that TFs play crucial roles in plant signal regulatory networks and control plant growth and root development, as well as responses to abiotic stress, *TaMYB20* gene is hypothesized to influence plants’ root system architecture (RSA) and improve their adaptations to osmotic stress and oxidative damage. According to Janiak et al. [21], MYB TFs are encoded by a wide range of genes that regulate primary and secondary metabolism, hormone synthesis and signaling, and stress response. Lateral root branching from the main root classes enhances the exploration intensity of minerals within the soil mass. *MYB96* enhances lateral root formation, although its transcript level in leaves and flowers is significantly greater than that in the lateral root primordia [4]; moreover, its overexpression enhances root branching through the activation of the well-known ABI3 and ABI5 genes, which are involved in the ABA signaling pathway. Our previous study showed that an adapted root in wheat (*Triticum aestivum*) was correlated with high antioxidant ability [22]. The R2R3-MYB *TaMYB20* was found to be a key factor in drought tolerance [23] and to display antioxidant activity preventing oxidative damage [19]. In the present research, we aimed to study the role of R2R3-MYB *TaMYB20* in maintaining the RSA traits in transgenic tobacco subjected to extended osmotic stress. Thus, our findings showed that *TaMYB20*-overexpressing tobacco lines exhibited enhanced osmotic stress tolerance via maintaining of ROS balance, preventing water loss, regulating auxin-related genes expression, and improving the root system architecture.

## 2. Materials and Methods

### 2.1. Plant Material and Osmotic Stress Treatments

Surface-sterilized *T. aestivum* cv. Sakha94 seeds (1% sodium hypochlorite solution for 20 min and washed twice with autoclaved double distilled water) were placed on filter paper in 9 cm petri plates containing 10 mL of half-strength MS [24] liquid medium at 25 °C for 5 days. When the seedling roots reached nearly 9 mm, they were transferred to a sterile magenta box, filled with sieved vermiculite, and grown for 10 days before osmotic stress was applied via 15% polyethylene glycol (PEG) 6000. The seedling roots were sampled at 0, 3, 6, 12, and 24 h, frozen in liquid nitrogen, and stored at −80 °C for RNA extraction.

### 2.2. Isolation of TaMYB20 Gene

The cDNA of *TaMYB20* was amplified via PCR using the primer pairs *TaMYB20*-F and *TaMYB20*-R (Table 1), which were designed using the NCBI primer design tool (https://www.ncbi.nlm.nih.gov/tools/primer-blast/ (accessed on 18 March 2023)). The accession (KY013614) was used as a template to design primers pairs. The cDNA templates were synthesized from RNA mixtures extracted from wheat seedlings roots that had been treated with 15% PEG 6000.

### 2.3. Semi-Quantitative RT-PCR

RNA extraction was performed using an RNeasy Plant mini kit (Qiagen, Germantown, MD, USA) according to the manufacturer’s protocol. First-strand cDNA was synthesized from the RNA of both wheat and tobacco using a QuantiTect Reverse Transcription Kit (Qiagen). The expression patterns of *TaMYB20* in *T. aestivum* were assessed in a reaction solution containing two specific primers (q*TaMYB20*-F and q*TaMYB20*-R; Table 1) and 2 µL of 1/10 dilution cDNA as a template. The reaction included an initial incubation at 94 °C for 5 min, then 40 cycles of 94 °C for 15 s, 55 °C for 40 s, and 72 °C for 45 s. As an internal control, a fragment of actin gene was amplified using the primers *Taactin*-F and *Taactin*-R (Table 1).

RT-qPCR was performed to assess the transcript accumulation of three auxin-related genes (*NtPIN1*, *NtPIN2*, and *NtPIN8*) (Table 1) in *TaMYB20*-transgenic lines and WT tobacco plants under control and osmotic stress conditions (15% PEG). Total RNA was isolated from WT plants and *TaMYB20*-transgenic lines using RNeasy Plant mini kit (Qiagen) according to the manufacturer’s instruction. First-strand cDNA synthesis and RT-qPCRs were conducted as described above. A melting curve analysis was performed to check the primer specificity. A fragment of the actin gene was used as an internal reference (Table 1). The relative expression accumulation was calculated according to the 2^−ΔΔCT^ method [25].

### 2.4. Plant Transformation

To generate transgenic tobacco plants, the *TaMYB20* coding sequence was cloned into the pCAMBIA2300 vector under the control of CaMV35S promoter. The pCAMBIA2300-*TaMYB20* construct was subsequently transformed into *Agrobacterium tumefaciens* strain *EHA*105. Transformation was accomplished using the *A. tumefaciens*-mediated leaf disk method [26]. Seven independent tobacco cv. *Xanthi* transgenic T1 lines were obtained, and their segregation on MS media supplemented with 100 mg·L^−1^ kanamycin was recorded (Table S1). The copy number of the transgene was calculated using a T-test (Table S1), and three independent resistant transgenic lines were selected according to their transgene copy number and were grown to obtain the T2 homozygous lines for further analysis. The expression level of *TaMYB20* gene in T2 homozygous lines was monitored using RT-PCR analysis.

### 2.5. Stress Assay Analysis of the Transgenic Tobacco Plants

The homozygous T2 seeds of transgenic lines (L1, L2, and L4) were used in two experiments designed to evaluate their root systems architecture to adapt under osmotic stresses. Seeds were surface-sterilized with 75% ethanol for 30 s and 0.15% sodium hypochlorite for 20 min, followed by two washes using sterile distilled water. The wild-type (WT) and transgenic seeds were surface-sterilized and germinated on MS or MSo media (MS supplemented with 15% PEG 6000) and incubated in a growth chamber with a light cycle of 16 h light and 10 h dark and day/night temperatures of 21–23/16–18 °C. The germination rate was recorded every 2 days for a period of 20 days.

For osmotic stress assays, tobacco seeds were surface-sterilized as described above. The seeds were subsequently sown on 1/2 Murashige and Skoog (MS) media and incubated in a growth chamber under the same conditions used for the germination test. After the seedling roots reached 150 mm long, they were transferred to MS and MSo (MS0 supplemented with 10% PEG 6000) and allowed to grow for 35 days. The WT and transgenic lines (TL) were sampled to analyze the RSA, the presence of ROS, oxidative damage, and antioxidant enzymes activity under control and osmotic stress conditions. For each biological replicate, four tobacco seedlings were counted per plate, and three plates were analyzed per each line.

### 2.6. RSA Trait Measurements for WT Plants and Transgenic Tobacco Lines

Seeds of WT and *TaMYB20*-transgenic tobacco lines were surface sterilized and germinated as described above. The plants were subsequently transferred into a sterilized magenta box filled with vermiculite. The WT and transgenic lines were irrigated with MS media for 15 days, divided into two groups, and were irrigated with MS media and MS supplemented with 400 mM mannitol as an osmotic treatment. Twenty days after treatment, the roots were carefully and thoroughly removed by extracting the plants from the vermiculite and rubbed between two paper towels to remove the excess of media from the surface of the roots. The extracted roots were placed in plastic boxes, stained with toluidine red dye overnight before scanning and scanned using a flatbed hp scanner (Scanjet, G2410, 1200 dpi). The photos were analyzed using WinRHIZO software (V5.0, Regent Instruments, Quebec, QC, Canada). Four RSA functional traits were selected, including total root length (TRL), lateral root number (LRN), root surface area (RSa), and root volume (RV).

### 2.7. Histochemical In Situ Detection of ROS, ∆Ψm, O^•−^ and H_2_O_2_

The root tips from the 35-day-old seedlings under both treatments were sampled firmly and tested for the overproduction of cellular ROS through observing the fluorescence intensity of the dye 2′,7′-dichlorofluorescin diacetate (DCF-DA, cat. no.: 251520, Sigma-Aldrich Chemical Co., St. Louis, MO, USA). DCFH-DA (0.25 µM) in 1x PBS buffer solution was used as described by Azab et al. [22]. After 30 min of incubation, the roots were washed twice with PBS buffer and imaged using a fluorescence microscope (Nikon Eclipse 80i, Tokyo, Japan) and with filter set 10 (excitation 485 nm; emission: 530 nm). To obtain an accurate result, the experiment was carried out three times.

Tracking the ∆Ψm changes in the WT and transgenic plants was carried out through monitoring the fluorescence intensity of specific dye, rhodamine (Rh123), as described by Saquib et al. [27]. The root tips of the WT and transgenic lines in both treatment groups were stained with 15 µM·mL^−1^ of Rh123 for 35 min at 37 °C in the dark, and visualization was performed using a fluorescence microscope (Nikon Eclipse 80i, Tokyo, Japan) at an excitation wavelength of 520 nm and an emission wavelength of 590 nm.

The production of superoxide (O^•−^) in the 60-day-old root apex was detected using the NBT staining method, in which a 300 µM nitro blue tetrazolium chloride (NBT; cat. No.: N5514, Sigma-Aldrich Chemical Co., St. Louis, MO, USA) was dissolved in 0.1 M Tris-HCl, 0.1 M NaCl, and 0.05 M MgCl_2_, pH = 9.5, and incubated for 2 h. The presence of O^•−^ is visualized as a blue coloration.

### 2.8. Measurement of RWC and Ion Leakage

The relative water content (RWC) was carried out by using three roots from each plate as one biological replicate, which were weighed to obtain their fresh weight (FW) and were then rehydrated in deionized water at room temperature until they were fully turgid after 24 h and subsequently weighed for second time to record the turgid weight (TW). Finally, the roots were dried at 75 °C for 72 h and then subsequently weighed to record the dry weight (DW). The relative water content (RWC) was calculated by applying the data of the three samples to the following Formula (1) [28]:

(1) $$\mathrm{RWC}\;\left(\%\right)=\frac{\mathrm{FW}-\mathrm{DW}}{\mathrm{TW}-\mathrm{DW}}\times 100$$

The ion leakage (IL) was investigated as described by Ben Romdhane et al. [29] with some modifications. First, fresh roots (100 mg) were chopped into pieces; each piece was 2 cm in length and immersed in 15 mL of distilled deionized water in test tubes. Afterwards, the pieces were incubated in a 35 °C water bath for 90 min to determine the electrical conductivity (EC1). After the samples were boiled for 15 min and cooled, the final EC (EC2) was measured. The leakage percentage was calculated by applying the Formula (2):

(2) IL (%)=EC1/EC2×100.

### 2.9. Assays of Antioxidant Enzymes Activity

The activities of antioxidant enzymes, including catalase (CAT), superoxide dismutase (SOD), and peroxidase (POD), were evaluated in 35-day-old seedlings under control and osmotic stress conditions. A mixture of root tips collected from transgenic and wild-type plants were grinded, after which enzyme activity was measured via spectrophotometry according to the protocol of commercially available kits (Cyman Chemical Company, Ann Arbor, MI, USA).

### 2.10. HPLC Analysis of the Phenolic Compounds of Wild and Transgenic Plants

HPLC analysis was performed using an Agilent 1260 Infinity 2 series quaternary pump with integrated in-line degasser (Agilent Technologies, Santa Clara, CA, USA). The separation was carried out using Eclipse C18 column (4.6 mm × 250 mm i.d., 5 μm). The mobile phase consisted of water (A) and 0.05% trifluoroacetic acid in acetonitrile (B) at a flow rate 0.9 mL/min. The mobile phase was programmed consecutively in a linear gradient as follows: 0 min (82% A); 0–5 min (80% A); 5–8 min (60% A); 8–12 min (60% A); 12–15 min (82% A); 15–16 min (82% A); and 16–20 (82% A). The multi-wavelength detector was monitored at 280 nm. The injection volume was 5 μL for each of the sample solutions. The column temperature was maintained at 40 °C [30].

### 2.11. Statistical Analysis

Data were analyzed using XlSTAT, and the significant differences among tested tobacco lines were determined using Duncan’s multiple range test (*p* < 0.05). The data were presented as the means ± SD values of three biological replicates (n = 3).

## 3. Results

### 3.1. Expression Profiles of TaMYB20 in Wheat

**Figure 1:**
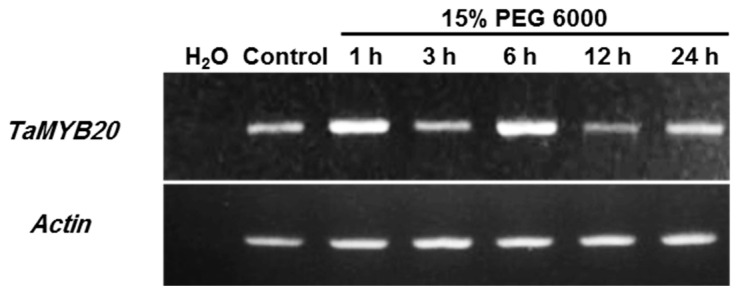
Expression pattern of TaMYB20 gene in *T. aestivum* plants subjected to osmotic stress. RT-PCR analysis was performed using TaMYB20-specific primers using the RNA isolated from the *T. aestivum* seedlings subjected to normal conditions (Control) and 15% PEG. Actin gene was used as an internal control (lower panel).

To understand the role of *TaMYB20* in the preservation of root system architecture traits under adverse environmental conditions, the accumulation of *TaMYB20* transcripts in response to osmotic stress was investigated in the roots of bread wheat cv. Sakha94 following exposure to high osmotic stress for 24 h. The expression level reached a high level after 6 h of treatment with 15% PEG 6000 and then declined to its normal level under control conditions, beginning at 12 h after treatment (Figure 1). These results confirmed that *TaMYB20* was upregulated by osmotic stress.

### 3.2. Isolation of TaMYB20 Gene and Generation of Transgenic Tobacco Plants

**Figure 2:**
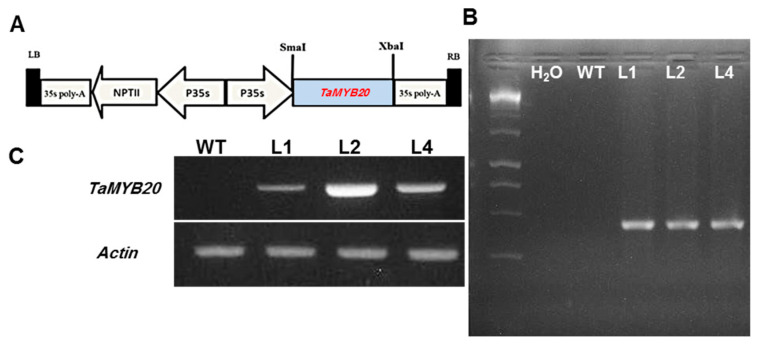
Molecular analysis of wild-type and *TaMYB20*-transgenic lines (L1, L2, and L4). Schematic map of the T-DNA region of the binary vector pCAMBIA2300:: *TaMYB20* (**A**). (**B**) PCR amplification of genomic DNA from wild-type (WT) and tree homozygous *TaMYB20*-transgenic lines. (**C**) RT-PCR analysis of *TaMYB20* expression level in WT and transgenic lines (L1, L2, and L4). Actin amplification was used as an internal control (lower panel).

The full-length cDNA clone *TaMYB20* was isolated using specific primers (Table 1) from stressed roots of *T. aestivum*. cv. Sakha94 treated with 15% PEG 6000 for 15 days. To investigate the physiological function of *TaMYB20*, seven transgenic tobacco lines were generated under control of the CaMV-35S promoter (Figure 2A).

Integration of *TaMYB20* and its expression into transgenic tobacco were confirmed by PCR and RT-PCR, respectively (Figure 2B,C). The copy number of transgene was evaluated via segregation analysis of *TaMYB20* gene in T1 plants to confirm the Mendelian inheritance pattern (Table S1). Based on *TaMYB20* transcript accumulation analysis (Figure 2C) three lines were selected for further assessment.

### 3.3. Evaluation of Stress Tolerance under In Vitro Conditions

**Figure 3:**
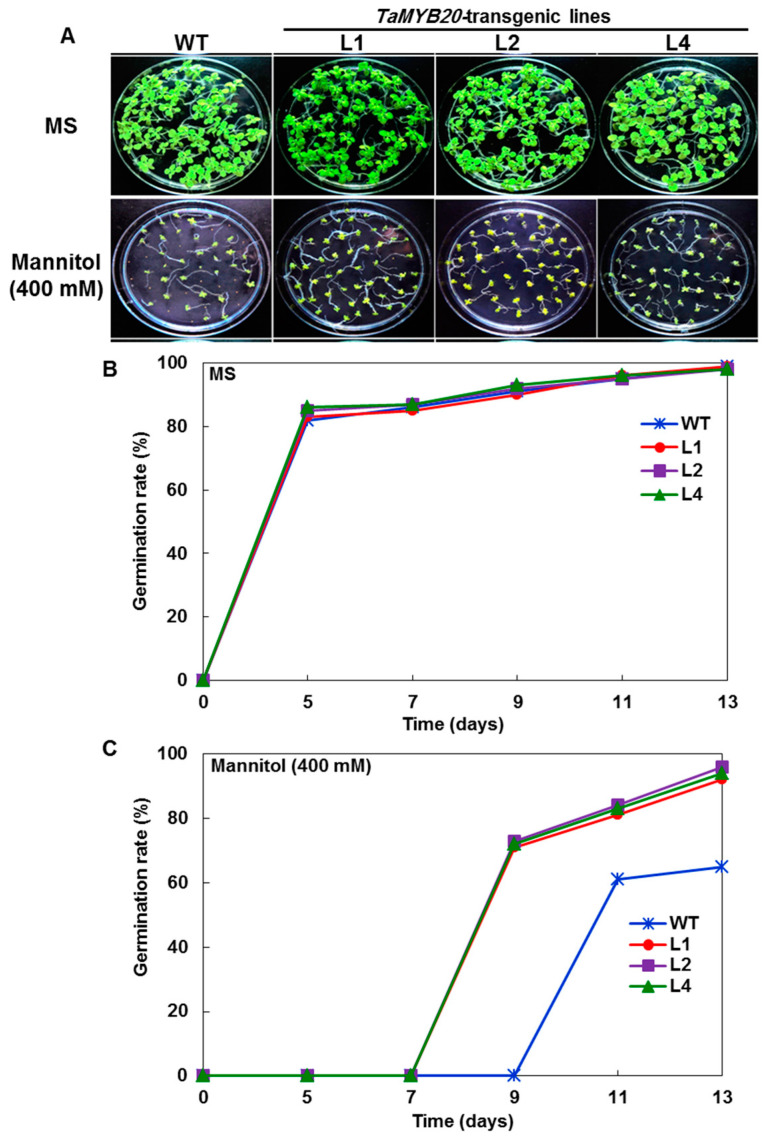
Effect of osmotic stress (400 mM mannitol) on seed germination. (**A**) Photos showed phenotypes of wild-type seedlings and transgenic lines after germination on MS media and MS containing 400 mM mannitol. (**B**) Seed germination rates of WT and transgenic lines on MS media (control) and (**C**) MS supplemented with 400 mM mannitol.

To assess osmotic stress tolerance, seeds of homozygous transgenic tobacco lines (L1, L2, and L4) and WT were germinated on MS media and MS supplemented with 400 mM mannitol. No significant differences in germination percentage were recorded for either the WT or transgenic lines under control conditions, in which the germination percentage reached 97% at 15 days post-germination (Figure 3A,B). However, after 20 days of 400 mM mannitol treatment, a significant decrease in the percentage of germinated seeds was detected for the WT compared to transgenic lines (Figure 3C). Notably, the germination of WT seeds was delayed until 9 days post-sowing, and 60% of seeds had germinated, while the germination of transgenic lines (L1, L2, and L4) was started after 4 days with a high percentage of germination, reaching up to 92% (Figure 3C). Furthermore, some of the WT seedlings ceased growing at the cotyledon leaf stage.

### 3.4. RSA Traits Measurements

**Figure 4:**
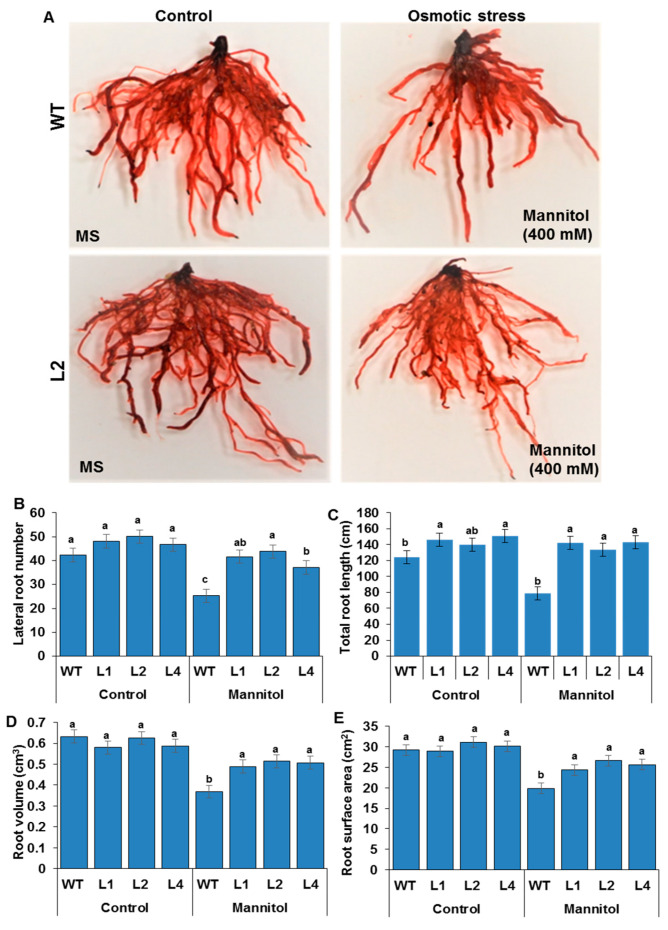
Tobacco plants grew under osmotic stress. Phenotypes of 35-day-old plants stained roots of wild-type and L2 tobacco seedlings grown in magenta box under normal and osmotic stress conditions (400 mM mannitol) (**A**). Quantification of lateral root number (**B**), total root length (**C**), root volume (**D**), and root surface area (**E**) in the wild-type and the three transgenic lines (L1, L2, and L4) under normal and 400 mM mannitol treatment. Values are means ± SD of three replicates. Different letters indicate significant differences at *p* < 0.05.

RSA is known to be strongly affected by stressful conditions. The ability of *TaMYB20* to improve RSA traits under drought stress conditions was assessed (Figure 4A). The four measured RSA traits (LRN, TRL, RV, and RSa) were affected by osmotic stress in both the WT and transgenic lines compared to those under control conditions (Figure 4B). With the progressive increase in osmotic potential in the magenta box, the RSA traits of WT plants were strongly affected and exhibited decreases ranging from 35% to 50% in the RV, RSa, LRN, and TRL. However, the *TaMYB20*-transgenic lines exhibited slight reduction (~10–15%) for all evaluated RSA traits compared to those registered under control conditions. Under osmotic conditions, the LRN and TRL traits displayed the highest reduction (~50%) in WT plants, while the transgenic lines showed ~10% reduction for these traits relative to those under control conditions. Interestingly, compared to control conditions, the transgenic lines displayed highest decline (~15%) in the RV trait under osmotic stress conditions (Figure 4B).

### 3.5. Expression of Auxin-Related Genes in TaMYB20 Tobacco Plants

**Figure 5:**
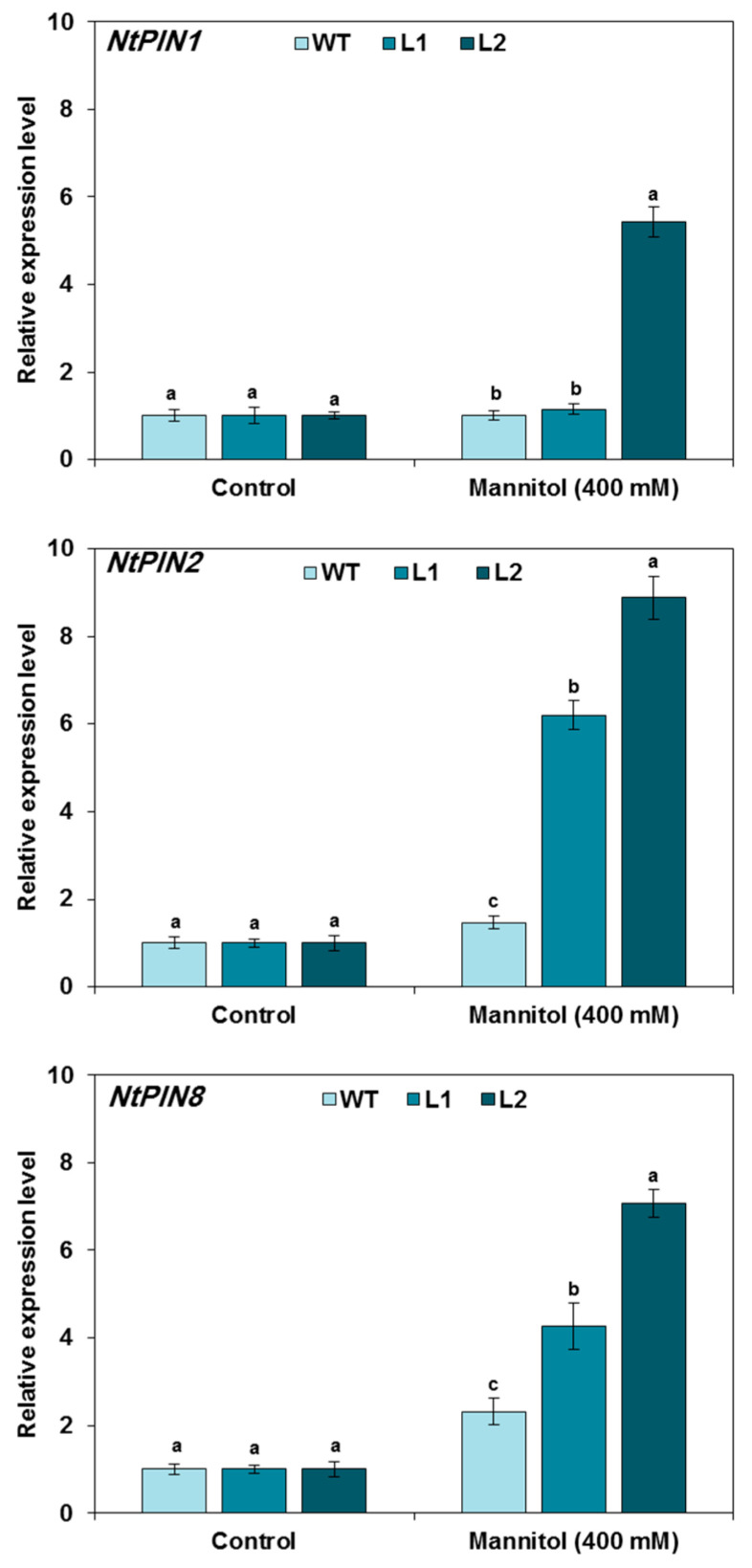
The expression levels of *PIN* genes in wild-type and transgenic tobacco seedlings subjected to control or mannitol treatment (400 mM). Values are means ± SEM (n = 3). Means sharing the same letter do not significantly differ at *p* < 0.05.

Auxin coordinates several plant growth and developmental processes, as well as adaptation to environmental stress. To gain further insight into the involvement of auxin-related genes in *TaMYB20*-mediated root system architecture (RSA) modifications upon osmotic stress conditions, the transcript accumulation of a subset of three auxin-related genes (*NtPIN1*, *NtPIN2*, and *NtPIN8*) was monitored in WT plants and two homozygous transgenic tobacco lines (L1 and L2) expressing *TaMYB20* at low and high levels, respectively. As shown in Figure 5, a slight regulation of the *NtPIN1*, *NtPIN2*, and *NtPIN8* transcripts accumulation was revealed in the transgenic lines (L1 and L2) compared to the WT plants under normal conditions. However, the L2 transgenic line, which strongly expresses the *TaMYB20* gene, showed a strong increase (~20-fold) into the expression of the *NtPIN1*, *NtPIN2*, and *NtPIN8* genes compared to WT plants under osmotic stress conditions. In addition, the expression patterns of auxin-related genes in the L1 transgenic line differ from those in the L2 transgenic line and are rather similar to those revealed in the WT plants under stress conditions; these findings may be due to the low expression level of the *TaMYB20* transgene in the L1 transgenic line.

### 3.6. Histochemical In Situ Detection of ROS, ∆Ψm, O^•−^ and H_2_O_2_

**Figure 6:**
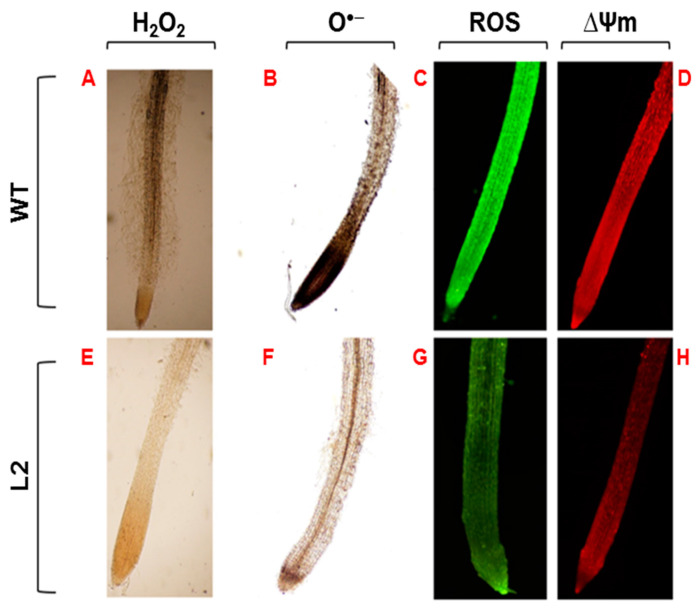
Oxidative damage of wild-type and L2 transgenic plants was assessed under osmotic stress (400 mM mannitol). Histochemical detection of H_2_O_2_, O^•−^, ROS, and ∆Ψm was performed using DAB, NBT, DCF, and Rh123 staining, respectively.

The decrease in RSA traits monitored under osmotic stress treatment prompted us to investigate oxidative damage and root affected zones through the localization of O^•−^ and H_2_O_2_. After staining, the roots subjected to osmotic treatment showed a differential distribution of all the stains in the root zones of WT and L2 transgenic line. However, the staining of the transgenic line roots (Figure 6E–H) was lighter than the WT (Figure 6A–D). Under osmotic stress, WT showed a spatial distribution of H_2_O_2_ in the elongation zone and for O_2_^•−^ in the root tip (Figure 6A,B). However, the transgenic line was able to scavenge these excess amounts (Figure 6E,F). The analysis of ROS status in WT and the transgenic lines revealed a non-typical pattern for both DCF and Rh123. The WT presented a sharp increase in fluorescence in the root tip and elongation zone, which suggested overproduction of ROS, affecting the oxidative status of root cells (Figure 6C) compared to those of the transgenic line (Figure 6C), which could tolerate the oxidative bursts and exhibited a marked reduction in DCF fluorescence.

To reaffirm the changes in ROS levels in the cell membranes of treated roots, the transgenic lines exhibited a discernable decrease in Rh123 fluorescence (Figure 6H), while an interesting increase in the fluorescence taking place in the root tip area of the WT plants (Figure 6D) was observed, possibly as a result of diffuse staining into the cytoplasm of the root cells.

### 3.7. Effects of Drought Stress on RWC and IL

**Figure 7:**
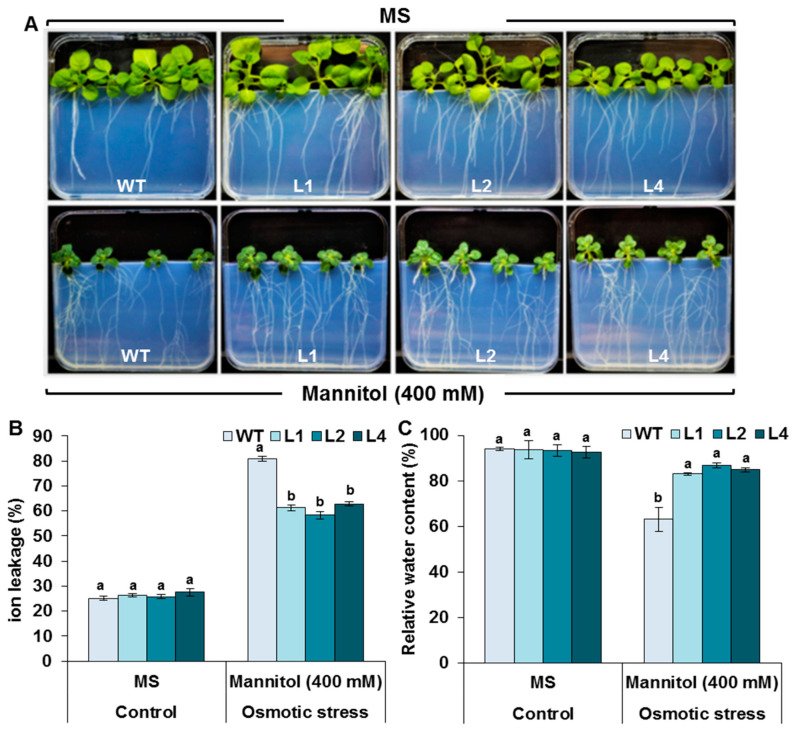
Effect of mannitol (400 mM) on RWC and IL in wild-type and transgenic lines. Fifteen-day-old tobacco seedlings grown under control conditions (in the upper row) or osmotic conditions (400 mM mannitol) (in the lower row). Values are means ± SD of three replicates. Different letters indicate significant differences at *p* < 0.05.

In order to investigate the drought tolerance of the transgenic plants, seedlings were exposed to extended osmotic stress (400 mM mannitol) for 35 days, which strongly altered plant growth (Figure 7A). The RWC and IL of the roots were calculated as important physiological parameters of drought tolerance to investigate the damage that occurred due to the lipid peroxidation of the plasma membrane causing ion leakage and a low percentage of RWC, as shown in Figure 7A–C. Marked changes in the RWC and IL were observed for the roots of WT and *TaMYB20*-transgenic lines after treatment with 400 mM mannitol. Compared with those of the transgenic plants, the roots of the WT plants exhibited severe IL release after osmotic stress (Figure 7B). Moreover, the RWC decreased for both the transgenic lines and the WT; however, compared with the WT, the transgenic lines showed a steep decrease in RWC, which decreased from 90% to 60% (Figure 7C). These results indicated that the overexpression of *TaMYB20* inhibited the effect of the oxidative burst caused by osmotic stress.

### 3.8. Overexpression of TaMYB20 Improves Antioxidant Enzymes Activities under Osmotic Conditions

**Figure 8:**
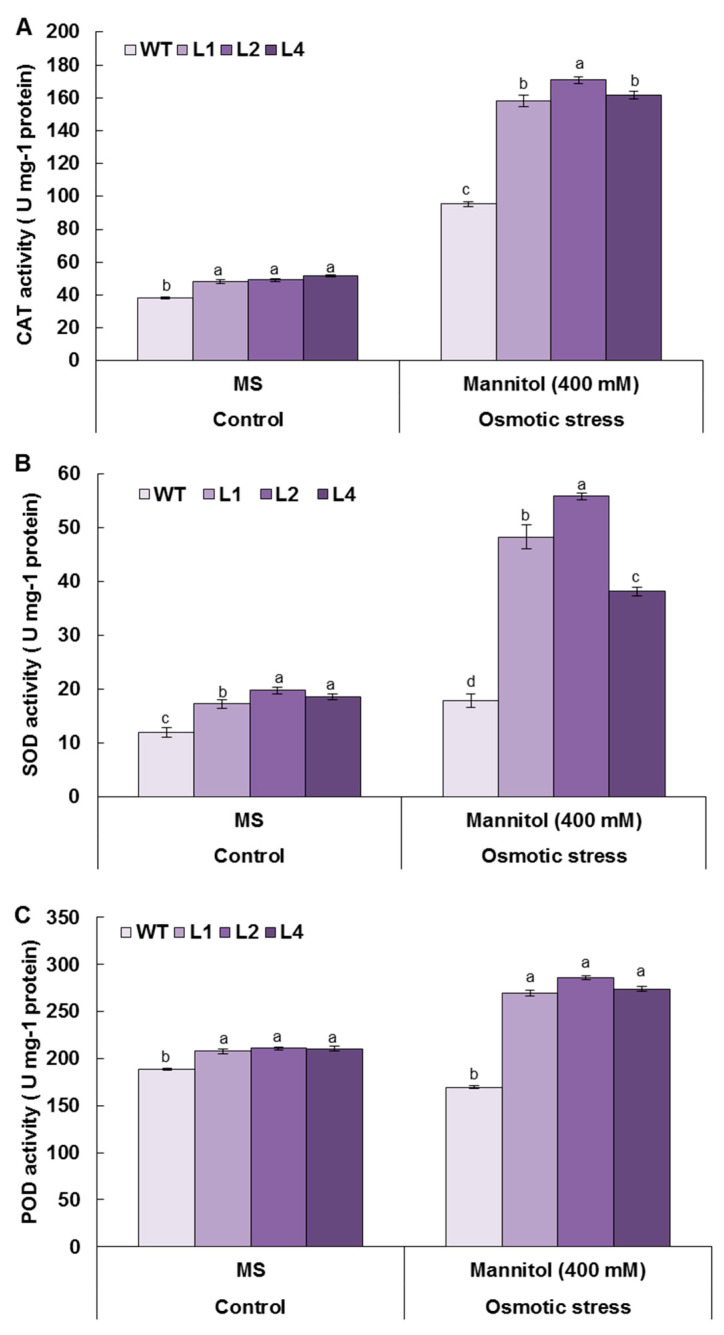
Activities of antioxidant enzymes (CAT, SOD, and POD) in tobacco seedlings subjected to mannitol treatment (400 mM). (**A**) CAT activity estimation, (**B**) SOD activity quantification, (**C**) POD activity estimation. Three independent biological replicates were used. Vertical bars refer to ± SD (n = 3). Different letters indicate significant differences at *p* < 0.05.

An efficient antioxidant enzymes activity can alleviate oxidative deterioration, which leads to enhanced drought tolerance. Thus, the antioxidant enzyme activities in the roots were measured after drought stress. The results indicated that transgenic lines exhibited higher levels of CAT, SOD, and POD activities compared to those in WT under osmotic treatment (Figure 8A–C). However, POD activity was not significantly different between the transgenic lines and WT plants under the control conditions (Figure 8C). After osmotic treatment, the CAT activity levels in the transgenic lines were greater than those in the WT (Figure 8A). Moreover, SOD enzyme activity levels were either significantly different between the transgenic lines and the control or within the transgenic lines under osmotic treatment (Figure 8B). However, POD enzyme activity increased in the transgenic lines but decreased in the WT plants after osmotic stress was imposed. These results confirmed the *TaMYB20* promotes the scavenging of oxidative bursts in plant cells through the activation of the antioxidant system under oxidative stress conditions.

### 3.9. Phenolic Compounds of Wild and Transgenic Plants

The phenolic compounds analysis of wild-type and transgenic tobacco plants revealed considerable variation the compound concentrations (Figure S1). In the wild-type tobacco, chlorogenic acid, coumaric acid, ferulic acid, daidzein, and quercetin were induced under stress conditions, while ellagic acid and hesperetin concentrations were reduced. The remaining compounds did not vary among the control or osmotically stressed tobacco plants (Figure S1A).

On the other hand, the two selected transgenic lines of tobacco plants showed considerable variability in response to osmotic stress (Figure S1B,C). In transgenic line 1 (L1), gallic acid, catechin, syringic acid, naringenin, and daidzein were induced under osmotic stress, while chlorogenic acid and vanillin concentrations were reduced (Figure S1B). Finally, compared with those in line 1, the response of transgenic line 2 (L2) was different from that of L1. In L2, the pyro catechol, ellagic acid, vanillin, ferulic acid, daidzein, quercetin, and hesperetin contents of tobacco plants were induced under osmotic stress, while the catechin, methyl gallate, and caffeic acid levels were reduced (Figure S1C).

## 4. Discussion

Data accumulation on the R2R3-type MYB gene from wheat is insufficient to identify the exact functions and mechanisms of these types of TFs. Overexpressed tobacco plants were generated to investigate the possible role of *TaMYB20* in RSA adjustment under osmotic stress. Transgenic lines exhibited obvious morphological differences in the RSA under osmotic stress, but they had a normal growth rate under control conditions (Figure 4A), which accords with previous results reported by Ma et al. [31] for TaMYB4. Additionally, *TaMYB20* transcript accumulation analysis revealed that it was upregulated in wheat plants subjected to osmotic treatment, which suggests its involvement in abiotic stress responses (Figure 1). Based on a previous transcriptomic analysis, Wei et al. [20] reported that *TaMYB20* is highly expressed in roots under drought stress conditions and can positively regulate many biochemical and physiological processes. Several previous studies on R2R3-type MYB gene family members reported that transgenic plants overexpressing members of this family showed enhanced osmotic stress tolerance [31,32,33]. Moreover, *TaMYB20-*transgenic plants exhibited greater plasticity of their root architecture under osmotic stress (Figure 4A,B) in addition to a marked increase in the number of lateral roots (LRN). Similar functions were reported for *AtMYB93* and *MYB36* in root primordium boundaries and lateral root formation in *Arabidopsis* [34,35]. By contrast, *AtMYB96* was responsible for the regulation of lateral root elongation but not responsible for lateral root formation [4], which explained the enhanced TRL in the transgenic lines compared to the WT plants subjected to osmotic stress (Figure 4B). Many articles have reported that the MYB family can be involved in the majority of biochemical and physiological processes in plants [21]. *TaMYB1*-overexpressing plants act as repressors of lignin biosynthesis [19], and a lower lignin content can promote cell elongation in roots; however, higher levels of lignin could be required to increase the mechanical solidity of developing roots under prolonged drought [23]. Overall, our findings suggest that *TaMYB20* could play a possible role in the regulation of several root traits under extended osmotic stress.

During osmotic stress, plants undergo cellular dehydration, leading to the disruption of cell membrane integrity [36]. Thereby, the plasma membrane becomes permeable, allowing the leakage of ions into the surrounding solution. Our analysis revealed that compared to those of WT plants, *TaMYB20*-transgenic lines exhibited lower ion leakage release and greater RWC after osmotic stress. These findings indicated that the overexpression of *TaMYB20* inhibited the effect of the oxidative burst caused by osmotic stress. In the same trends, *TaMYB30-B* from wheat [37], MYB22 from *Fagopyrum tataricum* [38], *OsMYB6* from rice [39], and GmMYB12 from soybean [40] reduced electrolyte leakage, as well as water loss rate, and improved RWC in transgenic plants.

Auxin is the key phytohormone that regulates root growth in plants [41]. Most auxin is synthesized in vegetative tissues (shoots and leaves) and then redistributed by auxin carriers, including PIN-FORMED (PIN) family proteins [42]. PIN proteins have been reported to be the most important polar auxin transporters and play a critical role in its distribution [43,44]. Previously, Xu et al. [45] reported that root emergence and development were significantly inhibited in *OsPIN1*-RNA interference transgenic *Oryza sativa* (rice) plants and suggested that *OsPIN1* plays an important role in auxin-dependent adventitious rice root emergence. Additionally, overexpression of *ZmPIN1* from maize in transgenic lines affects root development and growth. In rice, overexpression of *OsPIN2* led to the regulation of root elongation and lateral root formation via modulation of auxin distribution [46]. Recently, Lee et al. [47] revealed that *Atpin8-*mutant lines exhibit decreased lateral root density. In the present study, our findings showed upregulation expression of the *NtPIN1*, *NtPIN2*, and *NtPIN8* genes associated with root system architecture (RSA) modifications in the L2 *TaMYB20*-transgenic line compared with WT plants subjected to osmotic stress. Subsequently, these findings suggest that *TaMYB20* contributes to root system architecture establishment via the modulation of auxin transporter gene expression, which impacts plant osmotic stress tolerance.

The extended osmotic stress led to the overproduction and excessive accumulation of ROS, H_2_O_2_, and O_2_^−^ in the WT plants (Figure 6A–C), causing severe damage to plant membranes (Figure 6D), which was corroborated by lower RWC and greater IL in the WT roots than in the transgenic lines (Figure 7B,C). However, the transgenic lines were able to scavenge the overproduction (Figure 6E–G) and maintain better ∆Ψm (Figure 6H). It was reported that *TaMYB20* shares similar biological functions in the leaves of transgenic tobacco plants, where the *TaMYB20*-overexpressed plants showed a greater pattern of SOD and CAT enzyme activities [20]. Moreover, our results showed that CAT, SOD, and POD activities recorded greater levels in the roots of transgenic plants than in those of WT plants, confirming that *TaMYB20* activated the ROS-scavenging system in plant roots to mitigate oxidative damage.

## 5. Conclusions

In conclusion, a high-branched and extended RSA is considered a specific feature of drought-tolerant plants and is required for drought avoidance and improved water and mineral uptake. The overexpression of *TaMYB20* confers osmotic stress tolerance by increasing RWC and preventing water loss via the protection of root cell membranes. Moreover, TaMYB20 can boost the redox system of plants and regulate some auxin-related genes to maintain better root traits.

## Figures and Tables

**Table 1:** Primers for that used for isolation, transformation, and detection of the different genes.

Primers	Sequence 5′-3′
*TaMYB20*-F	CAGCGAACAGCGGCTCCCAAAAT
*TaMYB20*-R	GCACGGTCGTCGGGGTTTCCATT
*TaMYB20*-F	CCCGGGATGGGGAGGCAGCCGTGCT
*TaMYB20*-R	GCTCTAGATCACGGCCATGCTTCTTG
q*TaMYB20*-F	AAATTCCCGGGTCAGAAGGG
q*TaMYB20*-R	CATGCTTCTTGGTCGAAGCC
*Taactin*-F	AGTGGAGGTTCTACCATGTTTCCT
*Taactin*-R	CACTGTATTTCCTTTCAGGTGGTG
*TaMYB20*-F	CATGTACCTCCTCGGCATGG
*TaMYB20*-R	GGTAGTAGTGGTCGAACGGG
*NtActin*-F	TCCAGGACAAGGAGGGTAT
*NtActin*-R	CATCAACAACAGGCAACCTAG
q*NtPIN1c*-F	GCCTCCATTGCTGTTGGTCTA
q*NtPIN1c*-R	CAACAAAATGTAGTAAACCAAAGTGATA
q*NtPIN2*-F	AATAGTTTTGGAGGGGATGTTTTC
q*NtPIN2*-R	CCCCTTGTCTTCTTGTTGGTTC
q*NtPIN8*-F	TGACTTGGATCATAACAGGTCTTTC
q*NtPIN8*-R	AGATTCTTTAGTTGCATTGAGCTCA

## Data Availability

The data presented in this study are available in the article.

## Supplementary Materials

The following supporting information can be downloaded at https://www.mdpi.com/article/10.3390/biology13020128/s1: Figure S1. Concentration of different phenolic compounds within the wild and transgenic lines of tobacco plants either under control or osmotic stress (400 mM mannitol). Table S1. Segregation analysis of TaMYB20 gene in T1 generation progeny transgenic lines.
