# Supplementary material for: Ectopic Expression of a Wheat R2R3-Type MYB Gene in Transgenic Tobacco Enhances Osmotic Stress Tolerance via Maintaining ROS Balance and Improving Root System Architecture
Source: Biology (Basel). 2024 Feb 18;13(2):128. doi: 10.3390/biology13020128 (PMC10886976; doi:10.3390/biology13020128)
Supplement: Supplementary file 1 [file biology-13-00128-s001.zip › biology-2795675-supplementary.pdf]

Table S1. Segregation analysis of *TaODORANT1* gene in T1 generation progeny transgenic lines

Lines	Number of germinated seeds	Kanamycin (+)	Kanamycin (-)	% K+	% K-	Expected K+	Expected K-	Segregation Mendelian ratios	Calculated Chi-Square Value	Copies number
L1	47	34	13	72.34	27.66	35.25	11.75	3:1	0.18	1
L2	51	50	1	98.04	1.96	50.20	0.80	63:1	0.05	3
L4	51	45	6	88.24	11.76	47.81	3.19	15:1	2.65	2
L17	51	37	14	72.55	27.45	38.25	12.75	3:1	0.16	1
L21	50	38	12	76.00	24.00	37.50	12.50	3:1	0.03	1
L35	49	37	12	75.51	24.49	36.75	12.25	3:1	0.01	1
L50	49	37	12	75.51	24.49	36.75	12.25	3:1	0.01	1

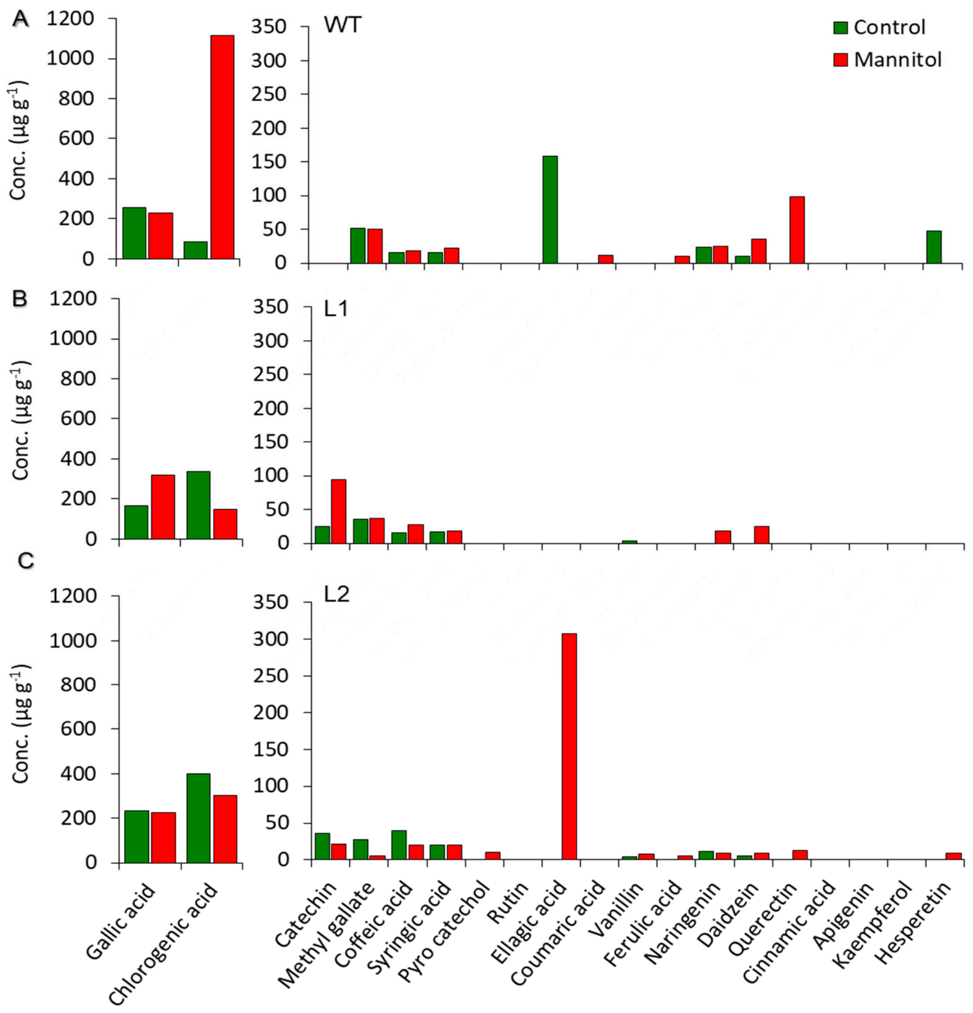

Figure S1. Concentration of different phenolic compounds within the wild and transgenic lines of tobacco plants either under control or osmotic stress (400 mM mannitol).
